# Dual single-cell and bulk RNA sequencing reveal transcriptional profiles underlying heterogenous host-parasite interactions in human peripheral blood mononuclear cells

**DOI:** 10.3389/fimmu.2025.1582645

**Published:** 2025-06-24

**Authors:** Praveena Chandrasegaran, Bekir Faydaci, Barbara Shih, Musa A. Hassan

**Affiliations:** ^1^ The Roslin Institute and Royal (Dick) School of Veterinary Studies, University of Edinburgh, Edinburgh, United Kingdom; ^2^ Division of Biomedical and Life Sciences, Faculty of Health and Medicine, Lancaster University, Lancaster, United Kingdom

**Keywords:** *Toxoplasma gondii*, host-pathogen interactions, PBMCs, monocytes, dendritic cells, single-cell RNA sequencing

## Abstract

*Toxoplasma gondii*, a zoonotic apicomplexan that infects over a billion people worldwide, can cause early death in immunocompromised individuals and defects in foetal brain development. *Toxoplasma* is also a major cause of abortion in small ruminants. When *Toxoplasma* encounters host cells, several outcomes are possible. For example, the parasite can enter the host cell or can inject its effector proteins into the cell without entering. These heterogenous outcomes occur simultaneously in the same host and likely determine disease pathogenesis. Yet, current knowledge of host-*Toxoplasma* interactions is largely based on averaged responses in bulk cell populations. Here, we employed single cell RNA (scRNA) and bulk RNA sequencing to investigate the transcriptional profiles that underpin heterogenous host-*Toxoplasma* interaction in human peripheral blood mononuclear cells. We observed that *Toxoplasma* preferentially infects and elicits transcriptional responses in dendritic cells in human blood. Additionally, we observed that monocytes adopt a dendritic cell-like transcriptional profile over the course of infection. Using genes expressed in sorted host cell populations representative of the different heterogenous host-*Toxoplasma* interaction outcomes as a reference panel, we show that genes expressed in cells infected via phagocytosis are largely expressed in dendritic cells. Thus, by integrating scRNA and bulk RNA sequencing, our study unveils the transcriptional profiles of diverse *Toxoplasma*-host cell interaction outcomes, providing novel avenues for targeted investigations into host gene functions during *Toxoplasma* infections.

## 1 Introduction

*Toxoplasma gondii* is a protozoan that infects virtually all warm-blooded animals (1, 2), including over a billion people worldwide (3, 4). When *Toxoplasma* interacts with phagocytic host cells, there are several possible outcomes. For example, the parasite can infect the cells via active invasion to form a parasitophorous vacuole (PV) or via phagocytosis to temporarily live in a phagosome (5–7). Besides infecting host cells, *Toxoplasma* can also inject its effector proteins into cells it does not invade (uninfected-injected host cells) (8). Thus, the outcome of *Toxoplasma* infection is a consequence of several heterogenous host-pathogen interaction outcomes that occur simultaneously within a host. For example, previous experiments showed that only human monocytes that phagocytose *Toxoplasma* secrete IL12 (7), which is needed to induce interferon gamma that is indispensable to effective host responses to *Toxoplasma*. However, other studies suggest that monocytes largely express CCL2 in response to the parasite (9). We propose that these variable observations are due to averaging heterogenous host responses in bulk cell populations and can be resolved by investigating host-*Toxoplasma* interactions at the single cell level.

Single cell RNA sequencing (scRNA-seq) is used to transcriptionally profile individual cells in composite cell populations (10). Previously, we used dual scRNA-seq to capture the transcriptome of both the host cell and *Toxoplasma* to reveal transcriptional segregation of human monocytes exposed to *Toxoplasma* for one hour (11), indicating that the transcriptional response to the parasite is driven by a subset of exposed cells. Nevertheless, the study was limited in scope as it involved only monocytes and a single infection time point. Here, we extended our previous study to understand the transcriptional programs that underpin heterogenous *Toxoplasma* interactions with human peripheral blood mononuclear cells (PBMCs) using a combination of dual scRNA-seq and bulk RNA-seq. We show that *Toxoplasma* preferentially infects dendritic cells (DCs) in human blood, triggering distinct transcriptional responses that vary not only between infected and bystander DCs but also among different DC subclusters. These findings suggest that *Toxoplasma* infection orchestrates a complex transcriptional modulation in DCs, influencing cellular responses and immunological signalling within the microenvironment. Additionally, our data show that monocytes transition to a DC-like transcriptional profile in response to infection, highlighting the potential adaptive changes in cell populations. By integrating scRNA and bulk RNA-seq, our study reveals divergent transcriptional responses within infected cells, and diverse roles of DCs during *Toxoplasma* infection.

## 2 Materials and methods

### 2.1 Isolation of human peripheral blood mononuclear cells

Whole blood was collected with written informed consent, according to the guidelines of, and with approval from, the Roslin Institute’s Health and Safety committee from healthy adult donors seronegative for *Toxoplasma*. PBMCs were isolated from whole blood using a standard Ficoll gradient protocol.

### 2.2 Parasites and infection

Tachyzoites of a type 1 *Toxoplasma gondii* (RH strain) were maintained by serial passage on human foreskin fibroblasts (HFFs). Parasites were grown in RPMI (Life Technologies) supplemented with 10% foetal bovine serum (FBS; Omega Scientific), 2 mM glutamine (Sigma), 10 mM HEPES (pH 7.5; Sigma), and 20 μg/ml gentamicin at 37°C in 5% CO_2_. For all infections, parasites were prepared by scraping heavily vacuolated HFFs, followed by syringe lysis. The released parasites were pelleted by centrifugation at 572 x g for 7 min, washed in phosphate-buffered saline (PBS; Life Technologies), filtered through a 5μm membrane to exclude host cell debris, and counted. The parasites were added to 2 x 10^6^ PBMCs in 6-well tissue culture plates at an intended multiplicity of infection (MOI) of 1, briefly centrifuged to bring the cells and parasites into contact and incubated at 37°C in 5% CO_2_ for 1h to allow infection. The cells were then rinsed twice in PBS to remove extracellular parasites and incubated in fresh RPMI media supplemented with 10% FBS at 37°C in 5% CO_2_ for 12h and 24h before processing for single cell RNA sequencing. Naïve uninfected cells were used as controls at each time point.

### 2.3 Single cell RNA library preparation and sequencing

Naïve (control), 12h, and 24h *Toxoplasma*-exposed human peripheral blood mononuclear cells from three healthy adult donors (*n* = 3 per group) were separately processed for 10X scRNA sequencing according to the manufacturer’s recommendation (10X Genomics 3’ version 3). Due to a lack of sufficient live cells, we were unable to sequence one of the 12h replicates. All scRNA-seq library preparations and sequencing were performed at the Institute of Genetics and Cancer (IGC) and the Wellcome Clinical Research Facility, respectively, at the University of Edinburgh.

### 2.4 scRNA-seq analysis

Raw scRNA-seq read counts were obtained using Cell Ranger v7.0.0 (12) based on the human (GRCh38, GENCODE) and *Toxoplasma* (ToxoDB-61) genomes and downstream analyses were performed in R 4.4.1 using Seurat v3 (13). Cells expressing less than 1000 genes and 1000 transcripts or more than 10% mitochondrial genes were excluded. Cell cycle scores (G2M and S scores) were estimated using canonical cell cycle marker genes obtained from biomaRt R package (14).

Normalisation was performed using SCTransform (15) by regressing out the effects of mitochondrial, ribosomal, and cell cycle genes before integrating the datasets. The number of principal components (PC) was determined using an elbow plot by plotting the PC standard deviation. Dimensionality reduction was performed with Uniform Manifold Approximation and Projection (UMAP) using the 20 PC and a resolution of 0.2.

### 2.5 scRNA-seq cluster annotation

The naive samples were subset and the markers for each cluster identified using the *FindAllMarkers* function in Seurat (**Supplementary Table 1**). Cell types were determined by combining supervised and unsupervised cell annotation approaches. For the unsupervised method, R packages such as scType (16), Azimuth (13), and SingleR (17) were used based on their built-in cell type reference gene database. We used ‘*Tissue = Immune system*’ reference for scType, the ‘*Human PBMC*’ reference for Azimuth, and the following datasets for SingleR: *HumanPrimaryCellAtlasData, BluePrintEncodeData, DatabaseImmuneCellExpressionData, NovershternHematopoieticData, and MonacoImmuneData*.

DCs were subset and the *FindVariableFeatures, RunUMAP, FindNeighbors*, and *FindClusters* features in Seurat used to identify variable features and to re-cluster the cells. We then performed pathway analysis using Metascape (18) to determine biological pathways enriched in different scRNA-seq cell cluster. Pseudotime trajectory analysis was performed in Monocle3 (19) using default parameters.

### 2.6 Bulk RNA library preparation

Due to the difficulty to fluorescently sort sufficient PBMCs representing the different host cell-*Toxoplasma* interaction outcomes (e.g., actively invaded or uninfected), we used the human monocytic cell line THP-1, which has been shown to mimic primary monocyte response to *Toxoplasma* (9). THP-1 cells were exposed for 12h to GFP-expressing RH parasites pre-labelled with pHrodo Red, a dye that fluoresces red in acidic conditions. 10^5^ cells representing each of the three populations: uninfected cells (GFP^-^ pHrodo^-^), cells infected through active invasion (GFP^+^), and cells infected via phagocytosis (GFP^+^ pHrodo^+^) were then sorted in cytometry. *Toxoplasma* can inject its effector proteins into host cells without infecting them, in a process known to rely on the contact between the parasite and host cell (8). Therefore, we also used naïve cells separated by a transwell membrane from cells exposed to *Toxoplasma* to generate true bystander cells that are neither infected nor injected with parasite effector proteins but still exposed to the host cell or parasite effectors secreted into the cell culture media. RNAs from naïve (unexposed), transwell, and sorted cell populations were separately extracted using RNA extraction kits (Qiagen) and processed for bulk RNA sequencing.

### 2.7 Bulk RNA-sequencing analysis

Transcript level quantification was performed by pseudoaligning the reads to both the human (release 42, GRCh38.p13, GENCODE) and *Toxoplasma* (TgondiiGT1, ToxoDB-61) transcripts using kallisto v0.44.0 (20). The transcripts were summed to gene-level counts using only the human gene annotation file (gtf). Approximately ~261,000 transcripts and ~ 63,000 genes were analysed. Differential gene expression was performed using DESeq2 v1.44.0 R package (21) by comparing each cell sub-population (infection outcome) relative to all other sub-populations. Genes with an adjusted *p*-value < 0.05 and log_2_FC > 2 were considered significant. Multiple testing correction was done using the Benjamini-Hochberg method. Pathway analysis was performed in Metascape (**Supplementary Table 7**) (18).

## 3 Results

### 3.1 Cells expressing dendritic cell markers significantly increase in *Toxoplasma*-exposed PBMCs

**Figure 1:**
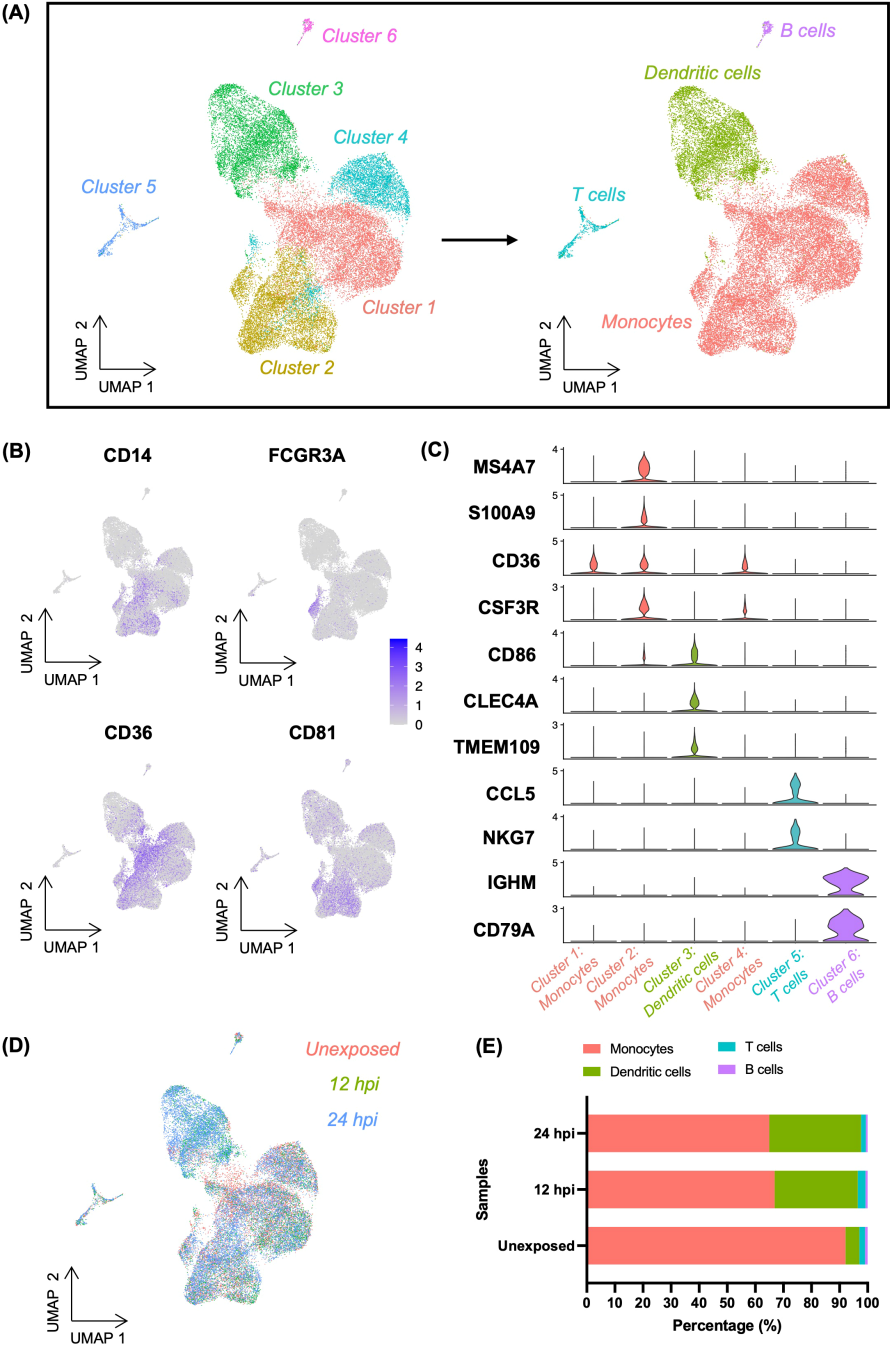
Classification of cells in *Toxoplasma*-exposed and unexposed human PBMCs. **(A)** UMAP projection of combined PBMCs at resolution 0.2, identifying six distinct cell clusters (left). Among these, three clusters were labelled as monocytes, which were further resolved into four distinct cell types (right). **(B)** Feature plots showing the expression of CD14 (classical monocyte marker) and FCGR3A (non-classical monocyte marker) as well as CD36 and CD81 (canonical monocyte markers) within the heterogenous monocytes cluster. **(C)** Violin plots showing the expression levels of cell type-specific gene markers across the four identified cell types. **(D)** UMAP depicting the distribution of cells categorised by experiment samples (Unexposed, 12hpi, and 24hpi) **(E)** Bar plot representing the percentage composition of each cell type across experimental samples.

PBMCs can provide a window into the complexity of the immune system and can be used to access the effect of different pathogens on the immune system. Thus, we characterised the transcriptional responses to infection in an heterogenous cell population by performing single cell RNA sequencing (scRNA-seq) analysis of human PBMCs exposed to *Toxoplasma*. To do this, freshly isolated PBMCs from three *Toxoplasma*-naïve donors were separately exposed to a clonal type I *Toxoplasma* strain (RH) at an intended MOI of 1 for 12h and 24h then processed for scRNA-seq analysis. Naïve (unexposed) cells were used as controls. Quality control, data normalisation, and doublets exclusion in Seurat v4.2.0 (Materials and Methods), yielded a total of 31016 high quality cells from the three donors, of which 10887, 6438, and 13691 were from the control, 12h, and 24h samples, respectively (**Supplementary Table 2**). Control, 12h and 24h cells had a median of 2595, 2558, and 2349 genes per cell, respectively (**Supplementary Table 2**). Unsupervised dimensionality reduction, clustering, and visualisation with uniform manifold approximation and projection (UMAP) identified six clusters (**Figure 1A**). Among these three clusters represented a heterogenous mix of monocytes (*MS4A7*, *S100A9*, *CD36*, *CSF3R*, *CD81*), including classical monocytes (*CD14*) and non-classical monocytes (*FCGR3A*) (**Figures 1A–C**). The remaining three clusters were identified as dendritic cells (*CD86*, *CLEC4A*, *CD81*), T cells (*CCL5*, *NKG7*), and B cells (*IGHM*, *CD79A*), based on the expression of known cell type specific markers (**Figures 1A, C**). All clusters contained cells from each donor, indicative of an absence of significant donor effects (**Supplementary Figures S1**, **S2**, **Supplementary Table 3**). However, the cell type compositions, particularly dendritic cells (DCs), changed upon *Toxoplasma* exposure (**Figure 1D**). Cell expressing DC gene markers increased from ~5% in control (unexposed) to ~30% and ~33% in 12h- and 24h-*Toxoplasma*-exposed cells, respectively (**Figure 1D**, **Supplementary Table 4**). The increase in cells expressing DC markers was accompanied by a decrease in cells expressing monocyte markers from ~92% in control (unexposed) to ~67% and ~65% at 12h- and 24h post-exposure cells, respectively. No significant changes in cell population were observed for the other cell types identified in the scRNA-seq data. This suggests that monocytes might acquire a DC-like marker expression phenotype in the course of *Toxoplasma* infection of human blood.

### 3.2 Heterogenous transcriptional responses and *Toxoplasma* burden across dendritic cell subclusters

**Figure 2:**
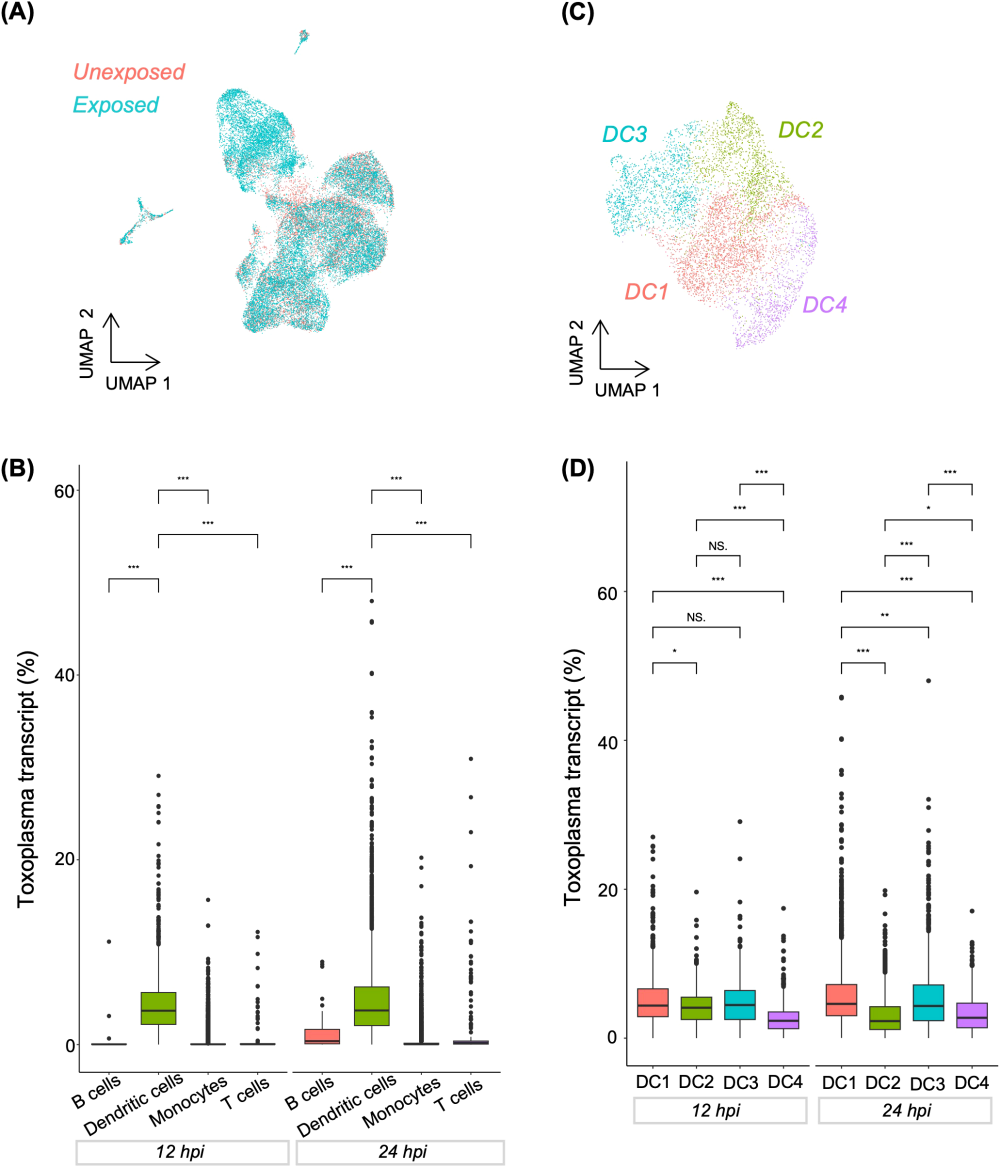
Distribution of *Toxoplasma*-infected cells in combined PBMCs and dendritic cell sub-clusters. **(A)** UMAP showing the distribution of cells categorised by experimental group (Unexposed and exposed). **(B)** Boxplot showing *Toxoplasma* transcript abundance across major immune cell types at 12 and 24hpi. **(C)** UMAP highlighting four sub-clusters within dendritic cell population. **(D)** Boxplot showing *Toxoplasma* transcript abundance across dendritic cell sub-clusters at 12 and 24hpi. **(B, D)** A Kruskal-Wallis test was used to assess differences in *Toxoplasma* transcript within each exposure group. Pairwise comparisons between cell types at each time were performed using the Wilcoxon rank-sum test. Asterisks denote statistical significance: ***p < 0.001, **p < 0.01, *p<0.05, and NS, not significant.

Previously, we reported that a subset of CD16^-^ monocytes drives the transcriptional response in monocytes exposed to *Toxoplasma* for 1h (11). Here, we sought to determine whether, in the presence of other myeloid cell types, monocytes are still the dominant cell type regulating transcriptional response to the parasite over time. Through an integrative analysis of the exposed and unexposed samples, followed by clustering and visualisation in UMAP, we identified DCs as the major cell type transcriptionally distinguishing *Toxoplasma*-exposed and unexposed cells (**Figures 2A, B**, **Supplementary Figure 3**). Previously, DC were reported to be the major myeloid cell type responding to *Toxoplasma*, based on the secretion of IL12, in human blood (7). Thus, we subset the exposure-distinguishing DC population and performed dimensionality reduction to investigate if the transcriptional response of DCs to infection was homogenous. We identified four transcriptionally distinct DC subclusters, DC1-4 (**Figure 2C**, **Supplementary Tables 5, 6**), suggesting heterogenous transcriptional response within the infection-distinguishing DC subset. Interestingly, we observed significant differences in the proportion of *Toxoplasma* transcripts across DC subclusters. At 12h post-exposure DC4 exhibited significantly lower parasite transcript levels compared to DC1-3, suggesting differential susceptibility to infection or parasite transcriptional activity in cells of the same cell type (**Figure 2D**). By 24hpi, all DC subclusters showed significant differences in parasite transcript abundance, indicating dynamic changes in infection burden or parasite transcriptional activity over time (**Figure 2D**).

**Figure 3:**
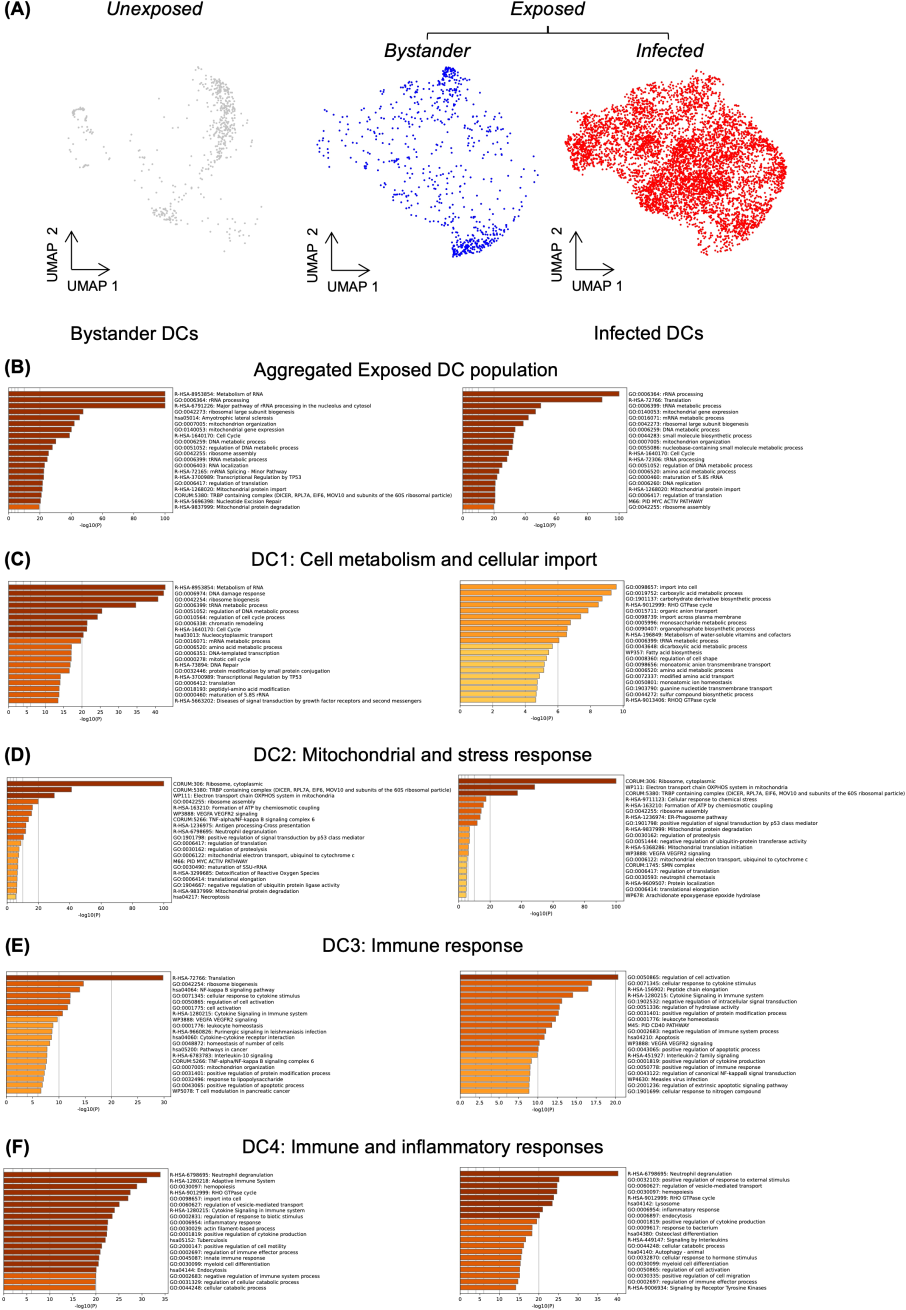
Differential biological pathway enrichment between dendritic cell sub-clusters. **(A)** UMAP showing unexposed and *Toxoplasma*-exposed dendritic cells. Within the exposed population, cells with less than 1% *Toxoplasma* RNA transcripts are classified as bystander cells, while those with more than 1% *Toxoplasma* RNA transcripts are classified as infected cells. Pathway enrichment analysis was performed exclusively on exposed cells using gene markers identified for bystander (left) and infected (right) dendritic cells **(B)** Analysis of aggregated exposed DC population. **(C-F)** Analysis of individual DC sub-populations (DC1-DC4), with pathway enrichment performed using Metascape.

Response to infection can be driven by infected and bystander cells. Thus, we investigated whether the transcriptional heterogeneity in *Toxoplasma*-exposed DCs was determined by “infected” or “bystander” status. To distinguish infected and bystander DCs, we used similarity in cell barcodes to match each infected cells with its cognate parasite RNA. We arbitrarily considered cells whose total RNA counts consisted of more than 1% *Toxoplasma* RNA transcripts as infected (**Figure 3A**). Pathway analysis performed for exposed DCs using genes expressed in infected and bystander cells revealed that both bystander and infected DCs shared enrichment in fundamental cellular processes, such as metabolism and cell cycle regulation (**Figure 3B**).

Differential pathway enrichment was assessed between infected and bystander DCs within each subcluster. In DC1, both bystander and infected cells showed similar enrichments, suggesting potential involvement in homeostasis and metabolic pathways (**Figure 3C**). In DC2, bystander and infected cells demonstrated strong overlap, with both groups enriched for mitochondrial functions and ribosome assembly pathways (**Figure 3D**). The shared enrichment may reflect a general stress response to *Toxoplasma* exposure. In DC3, both bystander and infected cells showed enrichment in cytokine-related and immune regulation pathways (**Figure 3E**). For DC4, bystander cells were enriched in pathways related to the adaptive immune response and cytokine signalling (**Figure 3F**). However, infected DC4 cells showed enrichment in lysosome and endocytosis pathways (**Figure 3F**), which may suggest an involvement in phagocytic activities. The differential responses observed across DC subclusters indicate complex transcriptional changes following *Toxoplasma* infection. These changes may reflect both immune activation and responses to infection. However, more target experiments are necessary to establish the functional relevance of these findings.

### 3.3 Monocytes shift to a dendritic cell-like transcriptional response during *Toxoplasma* infection

**Figure 4:**
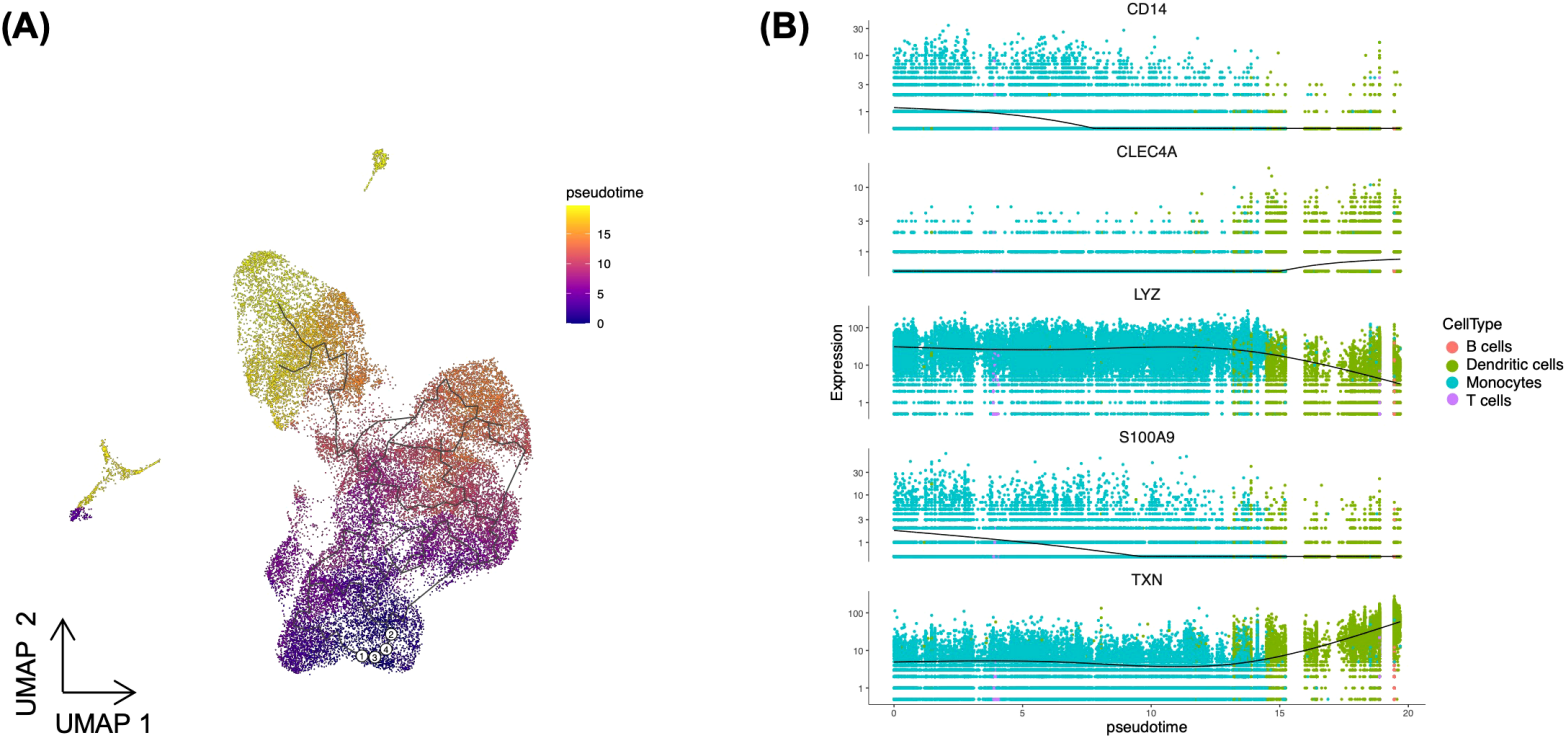
Pseudotime analysis illustrating dendritic cells differentiation from monocytes. **(A)** UMAP displaying the pseudotime trajectory of combined PBMCs. White circles (1-4) indicate root nodes representing classical monocytes, while black line depicts the trajectory. **(B)** Expression dynamics of dendritic cell markers (*CLEC4A* and *TXN*) and monocyte markers (*CD14*, *LYZ*, and *S100A9*) along the pseudotime trajectory. The black line shows smoothed average expression across pseudotime.

We observed a decline in cells expressing monocyte markers, which was accompanied by an increase in cells expressing DC markers. Thus, to investigate whether the transcriptional response of monocytes changes to DC-like response during long term exposure to *Toxoplasma*, we conducted a pseudotime analysis using Monocle3. Identifying this transcriptional shift could offer insight into how the immune landscape adapts during *Toxoplasma* infection. To establish the starting point of the pseudotime trajectory, we selected nodes representing classical monocytes, which serve as progenitors for various immune cell subtypes (22). We examined cells within the monocytes cluster to identify those expressing classical monocytes markers - *CD14^+^* and *FCGR3A^-^* (**Figure 1B**). These *CD14^+^FCGR3A^-^* cells were designated as the root nodes, establishing an origin point for tracking differentiation trajectories in the pseudotime analysis. Anchoring on these classical monocytes allowed us to map lineage-specific trajectories from monocytes to potential DC fates. The UMAP trajectory plot indicated a clear pathway from monocytes to DCs, suggesting that DCs could arise from the monocyte cluster over pseudotime (**Figure 4A**). To further validate this trajectory, we analysed the expression of monocyte- and DC-specific markers across pseudotime. Monocyte markers such as *CD14*, *LYZ*, and *S100A9* exhibited high expression at early pseudotime, predominantly localised within the monocyte cluster (**Figure 4B**). However, as cells progressed along the pseudotime trajectory towards DC fates, the expression of these markers gradually declined (**Figure 4B**). In contrast, DC markers, such as *TXN* and *CLEC4*, showed a progressive increase in expression along the trajectory, with their highest levels observed in differentiated DCs (**Figure 4B**). Overall, this pseudotime analysis supports a model where *Toxoplasma* infection induces a shift in monocyte transcriptional response towards a DC-like transcriptional profile. These DCs likely play a critical role in antigen presentation and pathogen clearance, thus, potentially serving as pivotal cells in shaping the host’s adaptive immune response to the infection.

### 3.4 Transcriptional heterogeneity is observed in *Toxoplasma*-exposed PBMCs

*Toxoplasma* interactions with phagocytic host cells can result in cells that are: *(i)* infected via active parasite invasion; *(ii)* infected via phagocytic uptake of parasite; *(iii)* uninfected but contact-dependently injected with parasite effectors (rhoptry proteins) (“uninfected-injected”); and *(iv)* neither infected nor injected (“uninfected-uninjected”). These heterogenous host-pathogen interaction outcomes, which occur simultaneously within a host, are potentially underpinned by distinct transcriptional programs. Although we were able to analyse individual host cell transcriptomes from the scRNA-seq, it was not possible to determine whether the transcriptional separation at the single cell level is driven by the heterogeneous host-parasite interactions. Thus, in an attempt at differentiating these heterogenous infection outcomes in the scRNA-seq, we separately sorted cells representing the different infection outcomes and performed bulk RNA-sequencing to obtain the corresponding reference genes. Briefly, THP-1, a human monocytic cell line that has previously been shown to mimic primary monocyte response to *Toxoplasma* (23, 24), was infected with type I (RH) *Toxoplasma* that constitutively express GFP and pre-labelled with pHrodo. Through FAC sorting, we obtained uninfected cells (GFP^-^ pHrodo^-^), cells infected via active invasion (GFP^+^), or phagocytosis (GFP^+^ pHrodo^+^) (Materials and methods). Because GFP^-^ pHrodo^-^ (uninfected) cells can consist of both “uninfected-injected” and “uninfected-uninjected” cells, we used cells in a transwell, which abrogates contact between THP-1 and *Toxoplasma*, to generate the “uninfected-uninjected” cells. Total RNAs from naïve (unexposed), transwell, and the different sorted subpopulation cells were extracted and separately processed for bulk RNA-sequencing.

**Figure 5:**
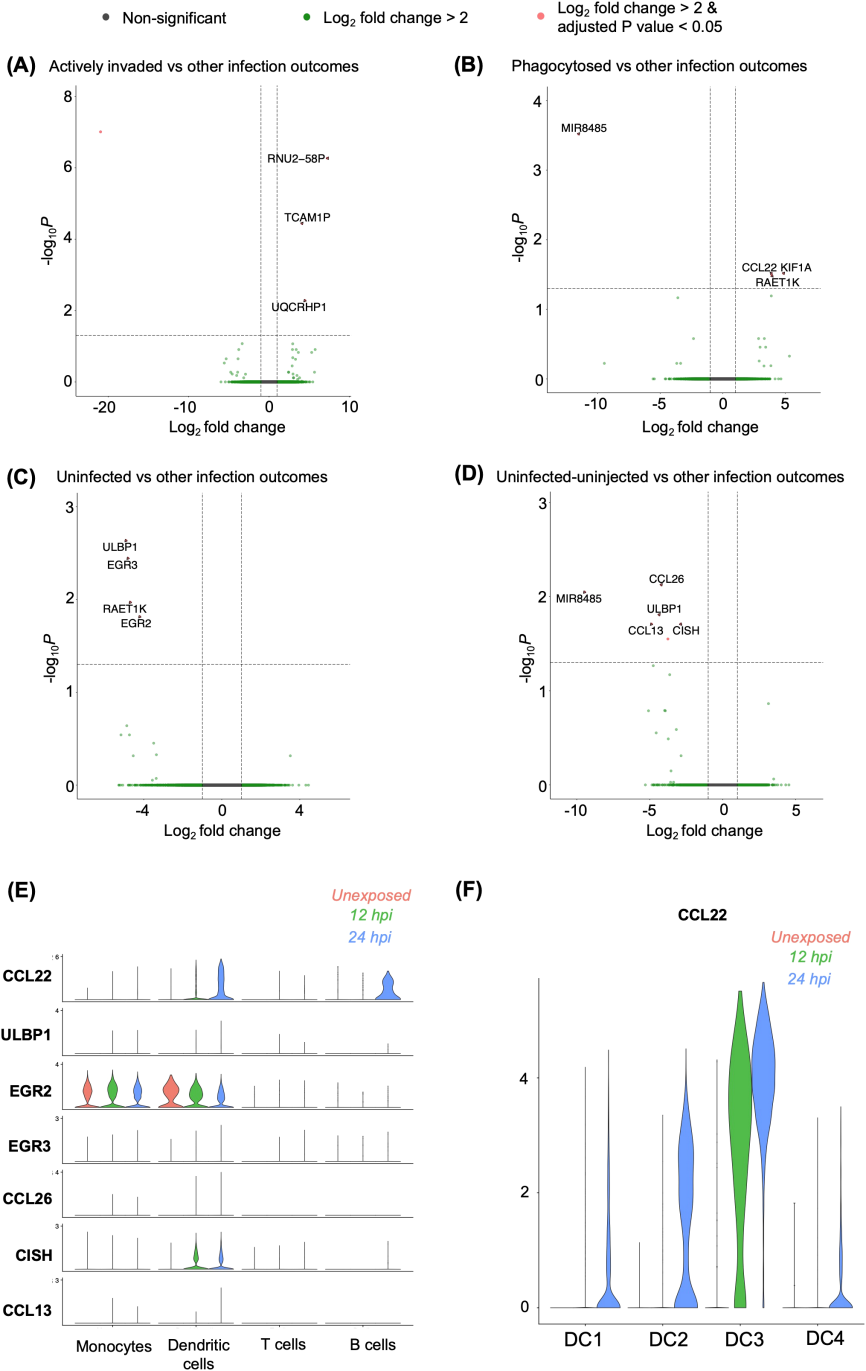
Differential gene expressions among heterogenous infection outcomes. **(A-D)** Differentially expressed genes identified in **(A)** actively invaded, **(B)** phagocytosed, **(C)** uninfected cells, and **(D)** uninfected-uninjected THP-1 cells compared to other infection outcomes, based on bulk RNA sequencing. Differential expression was determined using the criteria |Log_2_ fold change| > 2, and an adjusted *p*-value of <0.05. **(E)** Validation of differentially expressed genes identified in bulk RNA sequencing, shown across cell types in a single-cell RNA sequencing. **(F)** Expression of *CCL22* within dendritic cell sub-populations.

To identify a panel of reference genes for each sorted population, we performed a differential expression analysis (DEA) by comparing one sorted subpopulation against all other subpopulations. Based on this analysis, three genes (*RNU2-58P*, *TCAM1P*, *UQCRHP1*) were upregulated, and one (ENSG00000289901) was downregulated in the actively invaded group (GFP^+^) relative to the other infection outcomes: phagocytosed (GFP^+^ pHrodo^+^), uninfected group (GFP^-^ pHrodo^-^) and “uninfected-uninjected” group (transwell) (**Figure 5A**). Similarly, three (*CCL22*, *KIF1A*, *RAET1K*) genes were upregulated, while *MIR8485* was downregulated in the phagocytosed (GFP^+^ pHrodo^+^) population (**Figure 5B**). Rudzki et al. showed that secretion of *CCL22*, an immunomodulatory chemokine, is induced by *Toxoplasma* GRA28, which has potential involvement in parasite dissemination. *KIF1A*, a kinesin-like protein, is typically associated with neurological disorder (25). *RAETIK*, a long non-coding RNA, lacks a known function during infection. In contrast, four (*ULBP1*, *EGR3*, *RAET1K*, *EGR2*) genes were downregulated in uninfected cells (GFP^-^ pHrodo^-^) relative to the other three infection outcomes (**Figure 5C**). *ULBP1*, an NK cell receptor activator, is crucial for immunomodulation (26), while *EGR2/3* are early growth response genes essential for immune homeostasis (27–29). Additionally, five genes (*CCL26*, *MIR8485*, *ULBP1*, *CCL13*, *CISH*) and one unannotated gene (ENSG00000289505) were downregulated in the transwell relative to the other infection groups (**Figure 5D**). *CCL26* and *CCL13* are chemokines potentially involved in triggering immune responses. *CISH* is known to control cytokine signals in phagosomes during bacterial infection (30, 31).

To investigate whether different infection outcomes were overrepresented in individual cell clusters as defined by the scRNA-seq data, we analysed the expression of the differentially expressed genes from the sorted population in each cell cluster. None of the genes differentially expressed in the actively invaded group were expressed in the scRNA datasets (**Figure 5A**). One gene, *CCL22*, which is upregulated in phagocytosed group, was notably expressed in exposed DCs and B cells 24h post-infection (**Figures 5B, E**). Further analysis of the DC subclusters revealed that *CCL22* expression was predominantly observed in infected cells in the DC3 sub-cluster compared to the other subclusters (**Figure 5F**). Among the downregulated genes in uninfected group (**Figure 5C**), we noted that *EGR2* was expressed in both naïve and *Toxoplasma*-exposed monocytes and DCs (**Figure 5E**), whereas *ULBP1* and *EGR3* were lowly expressed in any cluster. Moreover, four genes (*ULBP1*, *CISH*, *CCL26*, *CCL13*) out of six downregulated genes in transwell were found to be lowly expressed in scRNA (**Figures 5D, E**). Thus, gene expression heterogeneity in *Toxoplasma* exposed PBMCs is largely not influenced by the heterogenous host-parasite interaction outcomes.

## 4 Discussion

Infection is driven by interaction between pathogens and individual host cells, resulting in different infection outcomes such as some cells becoming infected while others remain uninfected within the same individual. As such host-pathogen interactions are best studied at the single cell level. Here, we utilised a combination of dual scRNA-seq and bulk RNA-seq to *(i)* investigate the transcriptional profile of individual leukocytes during *Toxoplasma* infection, *(ii)* understand the transcriptional differences between bystander and infected myeloid cells, *(iii)* explore the origin and expansion of DCs in response to *Toxoplasma* exposure, and *(iv)* determine the transcriptional profile underpinning heterogenous *Toxoplasma*-immune cell interactions. Our data highlight the role of myeloid cells, especially DCs, in host response to acute *Toxoplasma* infection.

ScRNA-seq analyses revealed that DCs drive the transcriptional response to *Toxoplasma* in human blood and that monocytes adopt DC-like transcriptional profile during the course of *Toxoplasma* infection. These findings align with previous studies highlighting the critical role of DCs in controlling *Toxoplasma* infection in human blood (32–35). Previous studies have implicated monocyte-derived DCs in the pathogenesis of other parasitic infection. For instance, monocyte-derived DCs were postulated to induce T-helper cell responses to *Leishmania* infection (36). Similarly, monocyte-derived DCs are widely associated with inflammatory responses (37), where they are thought to contribute to both T-cell activation and innate immune responses (38). In this study, we observed increase in the proportions of cells expressing DC markers. This was accompanied by a decrease in the proportion of cells expressing monocyte markers. Subsequent pseudotime analysis showed the shift in expression of monocyte to DC markers during *Toxoplasma* infection. This monocyte-to-DC differentiation may represent a critical mechanism in mounting an effective immune response to the parasite. By sub-setting DCs, we did observed different infection patterns in DC subclusters. Our transcriptomic analysis further revealed that dendritic cell subclusters exhibit varying transcriptional profiles, with pathway enrichment analysis suggesting involvement in different biological processes across subclusters. This underscores the different responses of DCs to *Toxoplasma* infection, highlighting the complexity of host-pathogen interactions at the cellular level.

Although *Toxoplasma*-host cell interactions are known to simultaneously produce disparate outcomes, such as infected and uninfected-injected cells, the transcriptional programs underpinning these outcomes are equivocal. We identified differentially expressed genes in cell populations representing these different outcomes, including uninfected (cells that may have come into contact with the parasite but are not infected) and uninfected-uninjected (cells that are separated from the parasite by a transwell), highlighting potential transcriptional differences that could be missed when averaging responses across all exposed cells. Interestingly, only five differentially expressed genes in the different bulk cell populations were identified in the scRNA-seq data. This could be due to differences between THP-1, human monocytic cell line used in bulk RNA, and PBMCs used in scRNA-seq (39, 40). THP-1 contains only monocytes (40), which are professional phagocytic cells, while PBMCs have an assortment of immune cell types, including non-professional phagocytes. Additionally, while the transcriptional response in bulk cell populations is entirely based on THP-1 responses, the response in PBMCs is driven mostly by DCs. Several differentially expressed genes in both the bulk cell populations and single-cell clusters are known to modulate response to *Toxoplasma* infection. For example, the chemokine *CCL22* is known to be induced in cells that phagocytose *Toxoplasma* (41, 42). Interestingly, not all DC subclusters showed increased *CCL22* expression. Among the subclusters, only exposed cells within the DC3 subcluster exhibited increased *CCL22* expression. Within this subcluster, both infected and bystander cells showed enrichment in immune-related pathways. This, coupled with the increased *CCL22* expression, suggests that although the DC3 cluster is a dendritic cell sub-cluster, it may contain cells that phagocytose the parasite or that closely mimic the transcriptional response of monocytes that phagocytose *Toxoplasma*. Additionally, studies have demonstrated that *EGR2* is rapidly upregulated in infected cells in response to *Toxoplasma* secreted factors (43). *ULBP1* has been reported to control bacterial and viral infections (44, 45), suggesting its potential involvement in controlling *Toxoplasma* infection.

In conclusion, our scRNA-seq analysis provides insights into the diverse transcriptional responses of PBMCs during *Toxoplasma* infection. Dendritic cells appear to play a prominent role in the early immune response, with individual subpopulations exhibiting distinct transcriptional profiles. While comparisons with THP-1 cells offer some context, we acknowledge the limitations of comparing cell lines with primary cells. These findings underscore the importance of studying the host immune responses within diverse immune cell types. However, further experimental validation is needed to substantiate and refine the interpretations drawn from these computational analyses.

## Data Availability

The original contributions presented in the study are publicly available. This data can be found here: GEO accession number GSE295224 and Zenodo DOI https://doi.org/10.5281/zenodo.15240681.

## Supplementary material

Supplementary Figure 1 UMAP showing cell distribution by donor across experimental groups. Cells are coloured by donor to illustrate the distribution across the unexposed, 12 and 24hpi groups.

Supplementary Figure 2 Cell type composition across donors and experimental groups. Bar plot showing the percentage of each immune cell type for individual donors within the unexposed, 12, and 24hpi groups.

Supplementary Figure 3 UMAP depicting *Toxoplasma gondii* transcript abundance. The intensity of the colour indicates the percentage of *Toxoplasma* transcript detected, highlighting the distribution of infected cells.
