# Supplementary figures and images for: Dual single-cell and bulk RNA sequencing reveal transcriptional profiles underlying heterogenous host-parasite interactions in human peripheral blood mononuclear cells
Source: Front Immunol. 2025 Jun 24;16:1582645. doi: 10.3389/fimmu.2025.1582645 (PMC12234285; doi:10.3389/fimmu.2025.1582645)

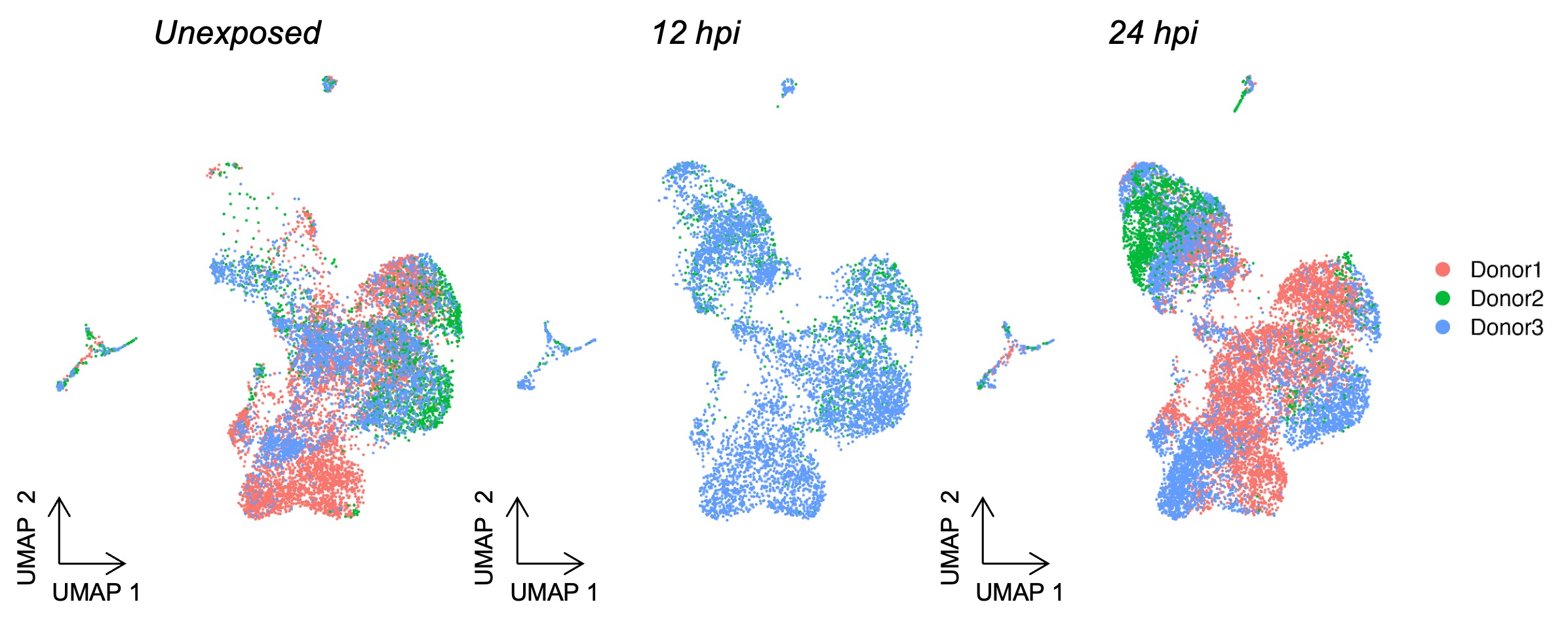

Supplement: Supplementary Figure 1 — UMAP showing cell distribution by donor across experimental groups. Cells are coloured by donor to illustrate the distribution across the unexposed, 12 and 24hpi groups. [file Image1.jpeg]

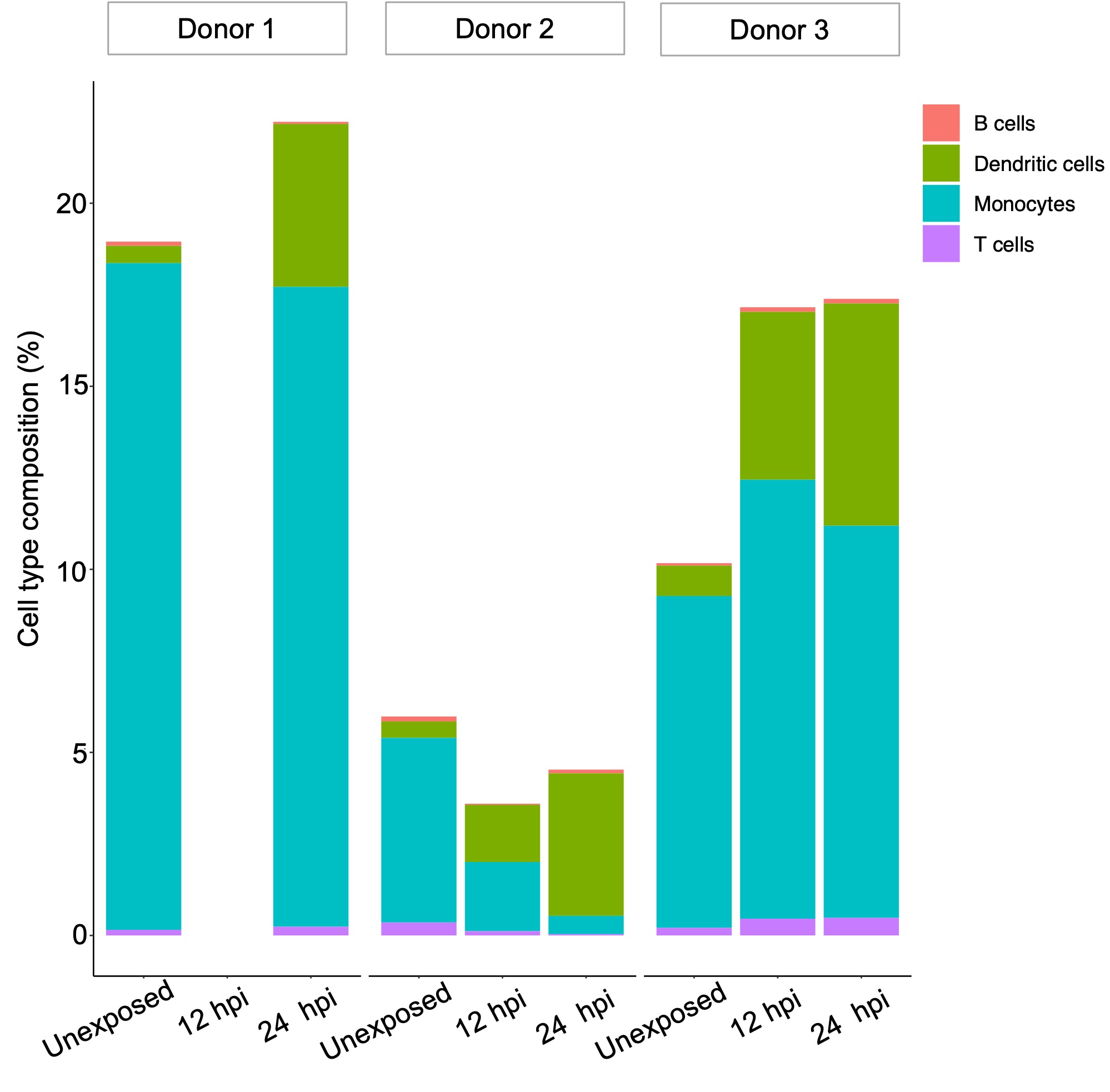

Supplement: Supplementary Figure 2 — Cell type composition across donors and experimental groups. Bar plot showing the percentage of each immune cell type for individual donors within the unexposed, 12, and 24hpi groups. [file Image2.jpeg]

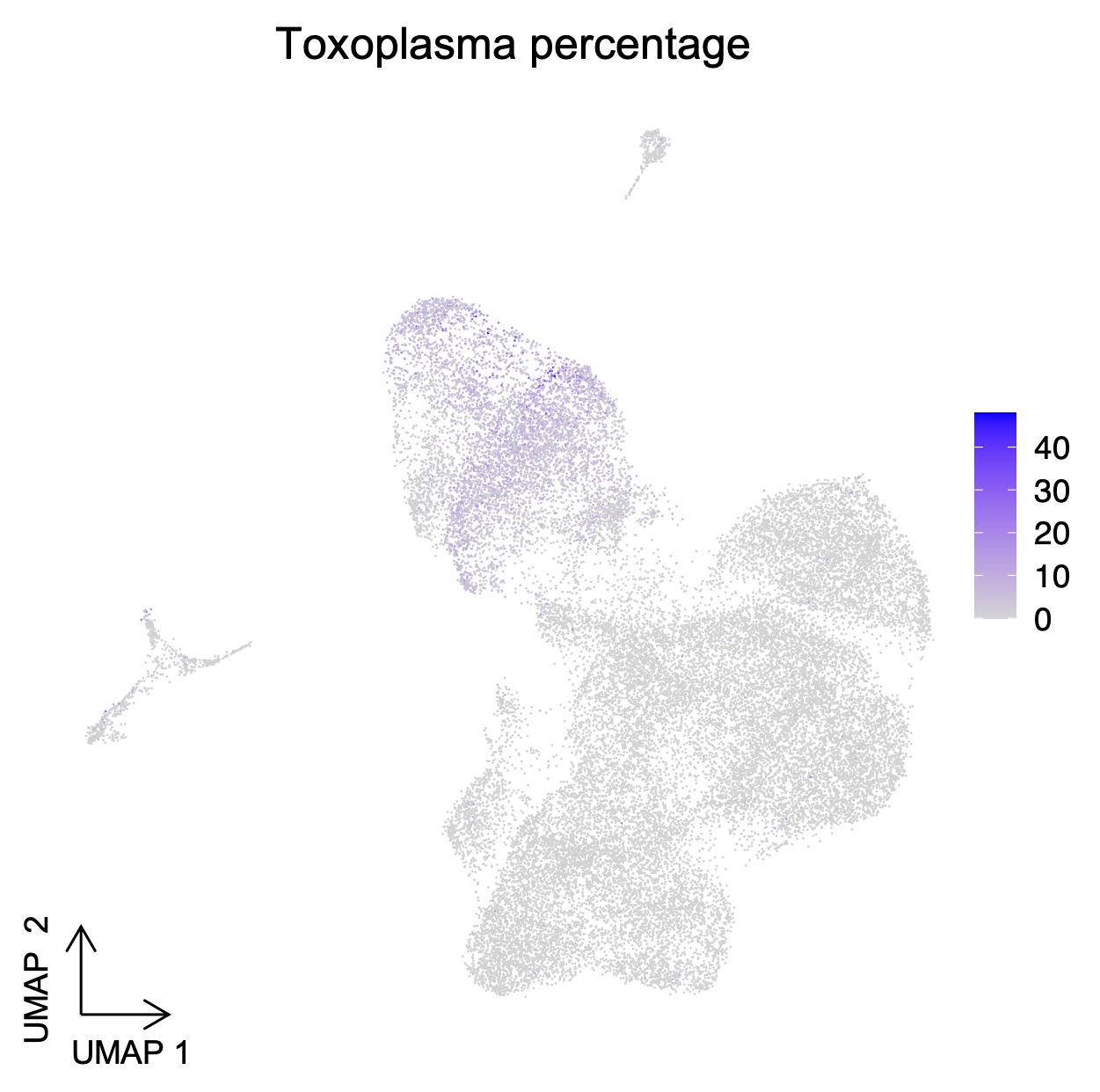

Supplement: Supplementary Figure 3 — UMAP depicting Toxoplasma gondii transcript abundance. The intensity of the colour indicates the percentage of Toxoplasma transcript detected, highlighting the distribution of infected cells. [file Image3.jpeg]
